# Machine learning models to predict submucosal invasion in early gastric cancer based on endoscopy features and standardized color metrics

**DOI:** 10.1038/s41598-024-61258-1

**Published:** 2024-05-07

**Authors:** Keyan Chen, Ye Wang, Yanfei Lang, Linjian Yang, Zhijun Guo, Wei Wu, Jing Zhang, Shigang Ding

**Affiliations:** 1 Department of Gastroenterology, Peking University Third Hospital, Beijing, 100191 China; 2 Beijing Key Laboratory for Helicobacter Pylori Infection and Upper Gastrointestinal Diseases (BZ0371), Beijing, 100191 China

**Keywords:** Early gastric cancers, Invasion depth, Color difference, Decision tree, Random forest, Gastrointestinal models, Oesophagogastroscopy, Gastric cancer

## Abstract

Conventional endoscopy is widely used in the diagnosis of early gastric cancers (EGCs), but the graphical features were loosely defined and dependent on endoscopists’ experience. We aim to establish a more accurate predictive model for infiltration depth of early gastric cancer including a standardized colorimetric system, which demonstrates promising clinical implication. A retrospective study of 718 EGC cases was performed. Clinical and pathological characteristics were included, and Commission Internationale de l’Eclariage (CIE) standard colorimetric system was used to evaluate the chromaticity of lesions. The predicting models were established in the derivation set using multivariate backward stepwise logistic regression, decision tree model, and random forest model. Logistic regression shows location, macroscopic type, length, marked margin elevation, WLI color difference and histological type are factors significantly independently associated with infiltration depth. In the decision tree model, margin elevation, lesion located in the lower 1/3 part, WLI a*color value, b*color value, and abnormal thickness in enhanced CT were selected, which achieved an AUROC of 0.810. A random forest model was established presenting the importance of each feature with an accuracy of 0.80, and an AUROC of 0.844. Quantified color metrics can improve the diagnostic precision in the invasion depth of EGC. We have developed a nomogram model using logistic regression and machine learning algorithms were also explored, which turned out to be helpful in decision-making progress.

## Introduction

The development of endoscopic submucosal dissection (ESD) techniques, which served as effective treatment towards early gastric cancers (EGC) has resulted in an improvement of survival quality. EGC is defined as gastric carcinoma which is limited to the mucosal (M) or submucosal (SM) layer of the stomach regardless of lymph node metastases. According to Japanese Gastric Cancer Treatment Guidelines^1^, the absolute indications for ESD include tumor size, ulceration, histological type, and infiltration depth. Among these, the accurate estimation of infiltration depth remains a clinical challenge.

In recent years scholars have researched applying conventional white-light endoscopy (WLI) to better estimate the infiltration depth and proposed different predictive features including macroscopic type, tumor size, lesion location, remarkable redness, margin elevation, uneven surface, enlarged fusion of converging folds and ulceration^2–5^. However, such existing models share inherent limitations, as their metrics are subject to empirical inaccuracy such as the definition of remarkable redness. For instance, developed by Abe et al^2^, DPS score which enrolled the variables including tumor size, margin elevation, remarkable redness and uneven surface was a widely used model with a specificity of 93.1–93.7%, but a relatively low sensitivity of 29.7–45.9%. Moreover, most of the endoscopic indicators are loosely defined which depend on the endoscopist’s experience. For example, the extent of remarkable redness is often subjectively qualified. Some studies have used the Commission Internationale de l’Eclariage (CIE) standard colorimetric system to standardize the color shade for the prediction of the infiltration depth of esophageal cancer^6^ which improves the visibility of colorectal polyps^7^ and the visibility of EGC after Helicobacter pylori eradication^8^, while yet being applied to the depth prediction of gastric cancer.

Although scholars have developed predictive models for infiltration depth using regression-based models, applying the findings directly to clinical scenarios remains challenging due to the intervened features in multi-dimension. To tackle the challenge, machine learning is adopted to analyze complex variable inter-relationships. Deep learning models like convolutional neural networks (CNNs), decision tree models and random forest models have shown great progress in medical fields^9–12^. CNNs are commonly used in the analysis of images. Yoon et al^10^ and Goto et al^9^ have developed diagnostic models of EGC using CNNs or using an artificial intelligence (AI) classifier, both achieving satisfying results. Although CNNs have some advantages in the recognition and processing of images, the interpretability of the model is poor compared to decision tree models and random forest models. The decision tree model has the advantage of capturing the non-linear relationship of more substantial features. Random forest models can further control the model variance, improve the accuracy, and estimate the importance of each variable. And to our knowledge, there are currently no studies using decision tree models and random forest models for gastric cancer infiltration depth prediction.

In this study, we not only included candidate predictors from previous studies but also innovatively introduced quantitative colorimetric indicators using CIE under WLI. A decision tree model and a random forest model were also developed to tackle the non-linearity challenges which achieved strong prediction accuracy and insightful clinical implication.

## Methods

**Figure 1:**
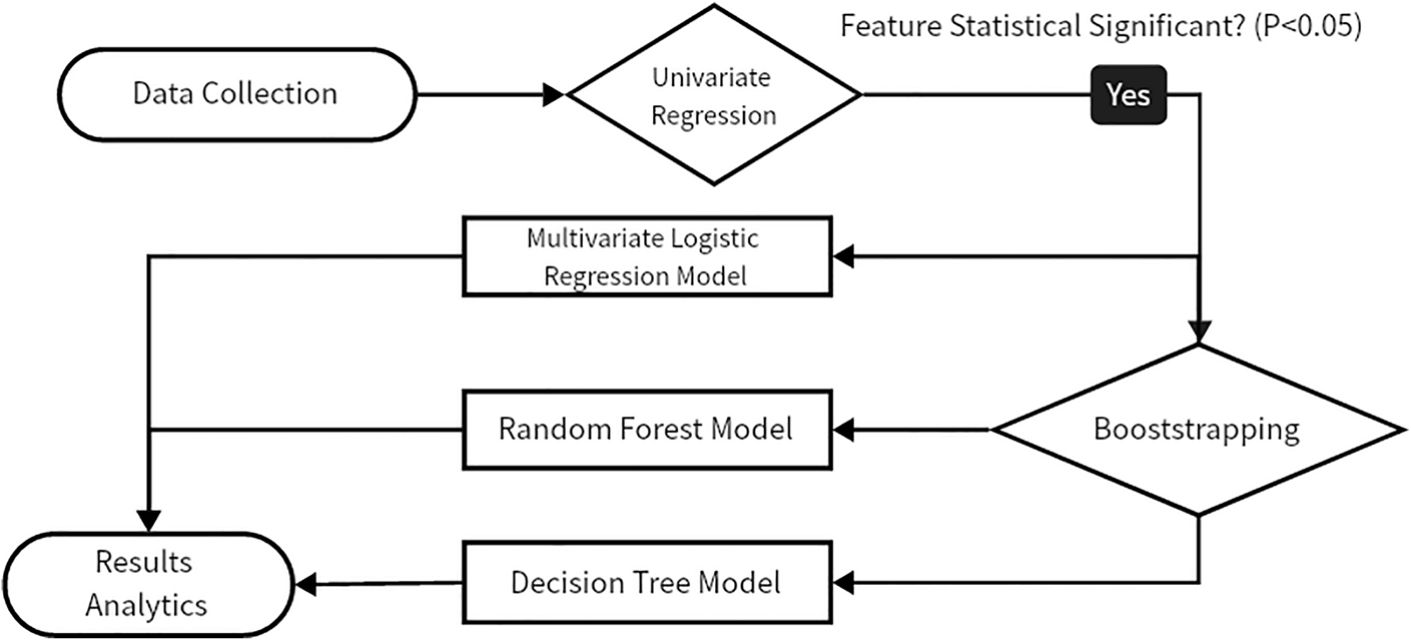
Research framework. After collecting the data, we performed a univariate analysis, selected the variables with significant differences (*P* value < 0.05), and performed a stepwise regression analysis while using bootstrapping to process the data to build a random forest model and a decision tree model.

The research framework is visualized in the flow charts (Fig. 1). Here we discussed the methodology in data processing and model design.

### Data selection

**Figure 2:**
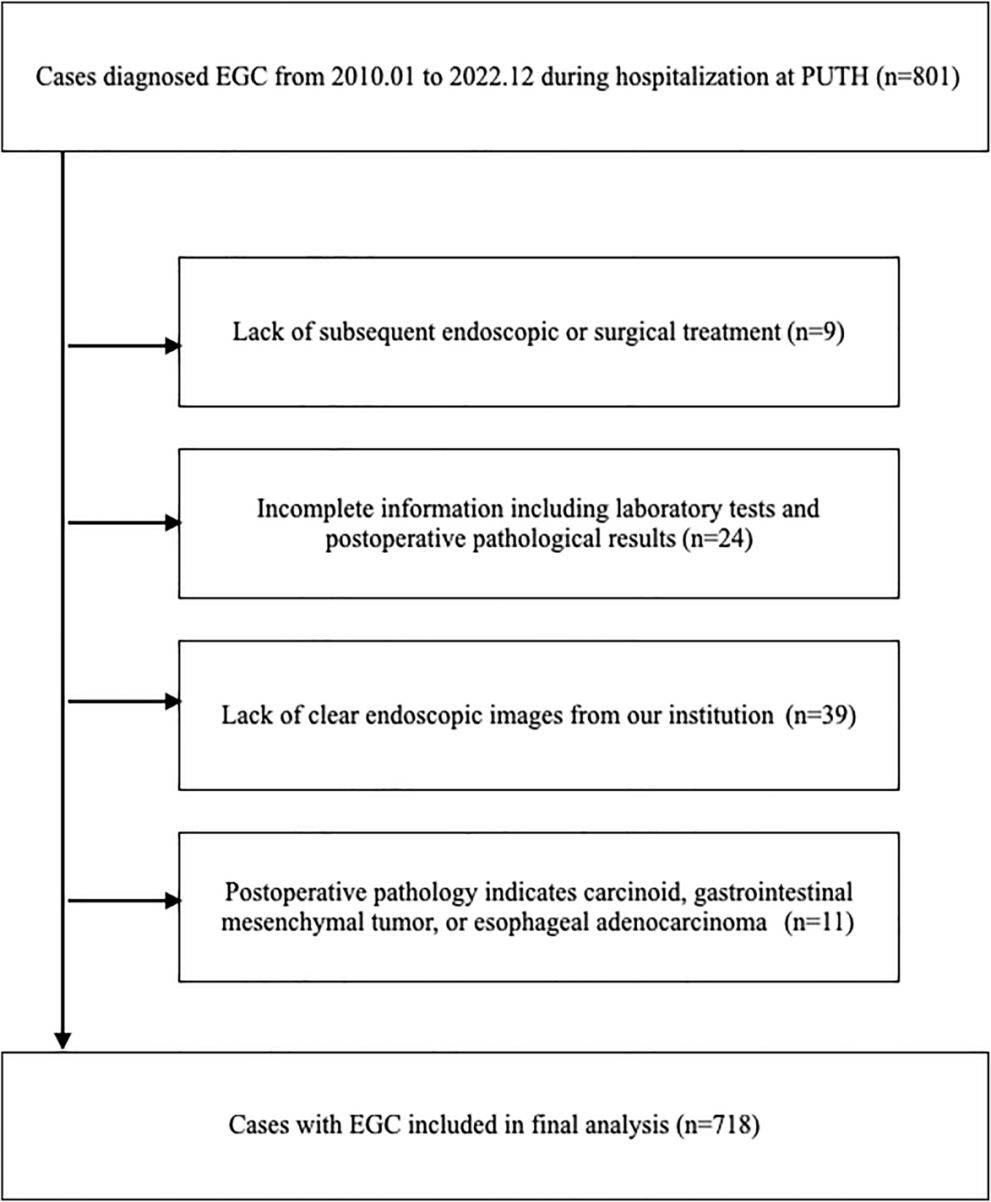
Study flow diagram and exclusion criteria.

A total of 801 early gastric cancer were diagnosed by pathological results from January 2010 to December 2022 during hospitalization at Peking University Third Hospital (PUTH). Endoscopy pictures were taken during routine procedures. 9 cases were excluded due to the lack of subsequent endoscopic or surgical treatment. 24 cases were removed due to a data loss ratio of more than 5 variables. 39 cases were excluded because of lacking clear endoscopic images from our institution. Clear images are defined as those with multiple angles including frontal and retroflexed angles, as well as clear of blood or debris. 11 cases were suspected of gastric cancer preoperatively, but postoperative pathology diagnosed other types of tumors such as carcinoid, gastrointestinal mesenchymal tumor, and esophageal adenocarcinoma. A total of 718 lesions were finally included in this study (Fig. 2). The study was approved by Peking University Third Hospital Medical Science Research Ethics Committee. All methods were carried out in accordance with relevant guidelines and regulations. Informed consent was obtained from all subjects or their legal guardians.

### Feature definition

Apart from the color shade, 11 other endoscopic features were selected as candidates for SM invasion predictors from the previous literature^2–4,13,14^ (Supplementary Fig. 1). The lesion diameter in this study was predicted endoscopically. The macroscopic type was classified into three categories: elevated type (I, IIa, and IIa + IIc), flat type (IIb), and depressed type (IIc, III, IIc + IIa). According to Nagahama’s study^13^, margin elevation was defined (1) as a manifestation of elevation of the lesion itself as a trapezoid elevation or (2) mucosal folds converged and were elevated at the lesion site when viewed from a distance of 15 to 45° under conditions of full extension of the gastric wall. Either a scar or an active area was defined as ulceration. Thickened or merged convergent folds were classified as enlarged folds. Endoscopic features were determined by reviewing endoscopic reports and under the guidance of experienced endoscopists. CT thickness is defined as an enhanced CT report of abnormal enhancement or thickening or visible tumor within the stomach or gastric wall.

### Histopathological staging

The endoscopically resected specimens were macroscopically sectioned at 4-µm intervals, processed for paraffin sections, and stained by hematoxylin and eosin (HE) by pathologists. As for the surgically resected specimens, pathologists select the deepest infiltration area and the surrounding area to exam unless the lesion is obscure, in that case the entire sample is sectioned as in the case of ESD. The invasion depth was determined based on postoperative pathologic findings, with those confined to the muscularis mucosae being classified as intramucosal carcinoma (group M), and those that extend beyond the muscularis mucosae to the submucosal layer but do not invade the gastric muscularis propria being classified as submucosal carcinoma (group SM). The degree of differentiated type was classified as differentiated type and undifferentiated type. Pure differentiated type (PD) was defined as those with more than 90% differentiated components in histology, pure undifferentiated type (PUD) as those with more than 90% undifferentiated components, and others were defined as mixed type. The degree of chronic atrophic gastritis is classified into 3 grades based on the reduction percentage of the intrinsic glands^15^. In mild degree cases, the intrinsic glands are reduced by about 1/3. It will be classified into severe degree when the intrinsic glands are reduced by more than 2/3. The rest is considered medium degree.

### Evaluation of color shade

**Figure 3:**
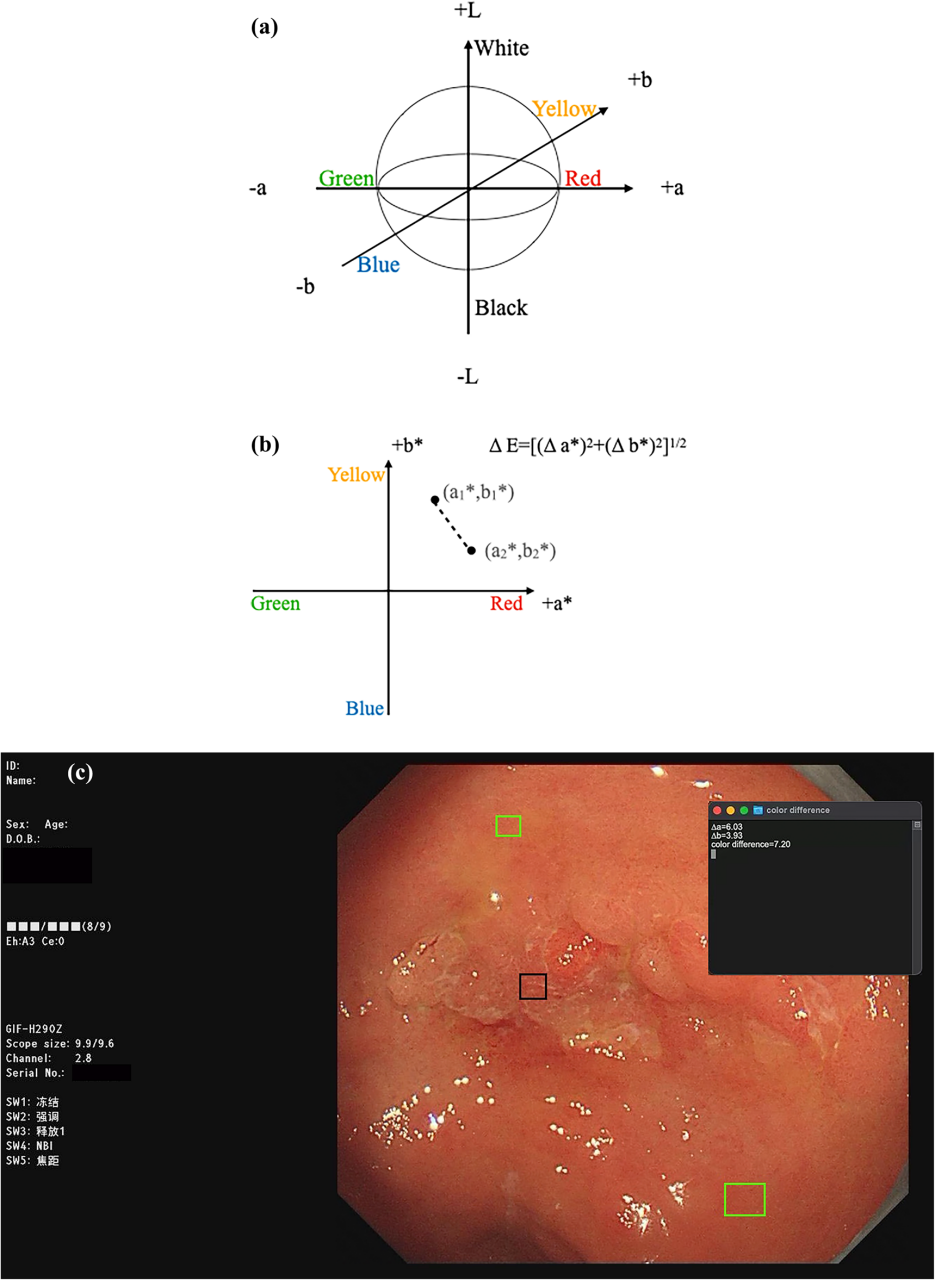
Calculation of color shade and color difference by CIE (L*a*b*). Color can be displayed in three-dimension (**a**). Setting L as a constant, the chromaticity is presented in two-dimension (**b**). And the color difference is calculated using the formula: ΔE = [(Δa*)^2^ + (Δb*)^2^]^1/2^. White light endoscopic schematic (**c**). The endoscopist selects the EGC lesion (black box) and the surrounding mucosa (green box), and the installed plug-in applet will randomly select the 5 sites in the black box to obtain their mean CIE values and select 2 sites in the green box to obtain their mean CIE value. The plug-in applet will automatically perform chromaticity calculations and display the color difference, Δa and Δb value.

The color difference between the carcinoma area and surrounding mucosal was calculated using the CIE (L*a*b*) color measurement methods, which demonstrates three-dimensional chromaticity, with the L-axis representing brightness, a-axis representing red-green spectrum, and the b-axis representing yellow-blue spectrum (Fig. 3a). The images were displayed in Photoshop to ensure a consistent measure of the L, a, b axis. Concerning the single displayed image, cancerous lesions were delimitated and subdivided into equal areas that are numbered and sites were selected through a random number generator. In total 5 sites were selected to obtain their CIE values and averaged them, while CIE values of 2 sites in the surrounding mucosa were obtained and averaged. Based on the methods of existing literature^6,16^ brightness was assumed to be constant, therefore L-dimension was excluded. A*color value and b* color value were two-dimensionally displayed (Fig. 3b). Color difference was calculated using the following formula: ΔE = [(Δa*)^2^ + (Δb*)^2^]^1/2^. The lesion observed under white light endoscopy using a plug-in applet is shown in the example picture (Fig. 3c).

### Feature preprocessing and statistical analyses

Categorical variables were presented as proportions, continuous variables were expressed as mean ± standard deviation. Samples were randomly grouped into derivation and validation set at the ratio of 7:3 using random number generator. Candidate predictors with a *P* value less than 0.05 were selected using univariate analyses, which were later included in backward stepwise regression analysis to identify independent predictors (*P* < 0.05). Data were analyzed Using R studio software, the rms package was applied to construct a nomogram model for predicting the depth of invasion of early gastric cancer based on logistic regression analysis.

### Bootstrapping

The original derivation set has 124 SM cases and 285 M cases, the resulting class imbalance will skew machine learning algorithms to the larger class (m in this study), which may bias the prediction. To mitigate the influence of the class imbalance, the bootstrapping method was adopted by randomly sampling up the positive cases to the same level of negative class.

### Decision tree methodology

To build a machine learning model with good interpretability, decision tree model was selected, which classified samples through a series of “feature split”. In every round of tree-growing, the split will be chosen on the nodes with the optimal feature and cut-off point that maximize the entire tree’s entropy decay. Both the decision tree model and the random forest model were developed using the open-source language Python3.7 and scikit-learn package.

### Random forest methodology

The single decision tree has its own advantage of intuitive model interpretation. However, the decision tree is sensitive to training samples and leans to overfitting, resulting in model variance. To control the variance, the bagging method was utilized to create a Random Forest model, which leveraged multiple decision trees and used majority votes to determine the final classification result. To ensure all samples and features will have chance to be evaluated, each single decision tree will be constructed using only a subset of samples and features to improve model performance.

## Results

### Baseline characteristics

A total of 718 lesions were enrolled in this study. Overall, demographically the age range of patients enrolled varied from 25 to 90 years, geographically covering 23 provinces in China, with a predominance of northern provinces. The mean (SD) age of patients was 63.56 ± 11.165 years. 216 (30.1%) were female and 502 (69.9%) were male. 439 lesions (61.14%) were resected endoscopically. Surgical resection was performed in 279 lesions (38.86%). According to the pathological results, there were 503 mucosal cancers and 215 SM invasions, respectively.

All cases were randomly allocated into a derivation set (n = 504) and a validation set (n = 214) with a ratio of 7:3. Supplementary Table S1 shows the clinical and pathological characteristics of both groups. Statistical test illustrated no significant differences between the two groups in age, gender, smoking history, alcohol consumption, gastric cancer history, laboratory findings, endoscopic characteristics, and enhanced CT results, while macroscopic types (*P* = 0.032) differ.

### Feature selection in statistical analyses

Univariate and Multivariate Logistic Analyses were performed to select the predictors. Univariate analysis (Supplementary Table S2) shows that patients with smoking history and a lesion located in the middle or lower 1/3 of the stomach, depressed macroscopic type, enlarged fold, marked margin elevation, nodular surface, ulceration, containing undifferentiated histological type, preoperative biopsy revealing cancer, larger color difference and higher a* value were associated with infiltration depth statistical significantly. WLI a*value and color difference cutoff points were set at 10.1 and 14.4 respectively with maximized Youden index.

**Figure 4:**
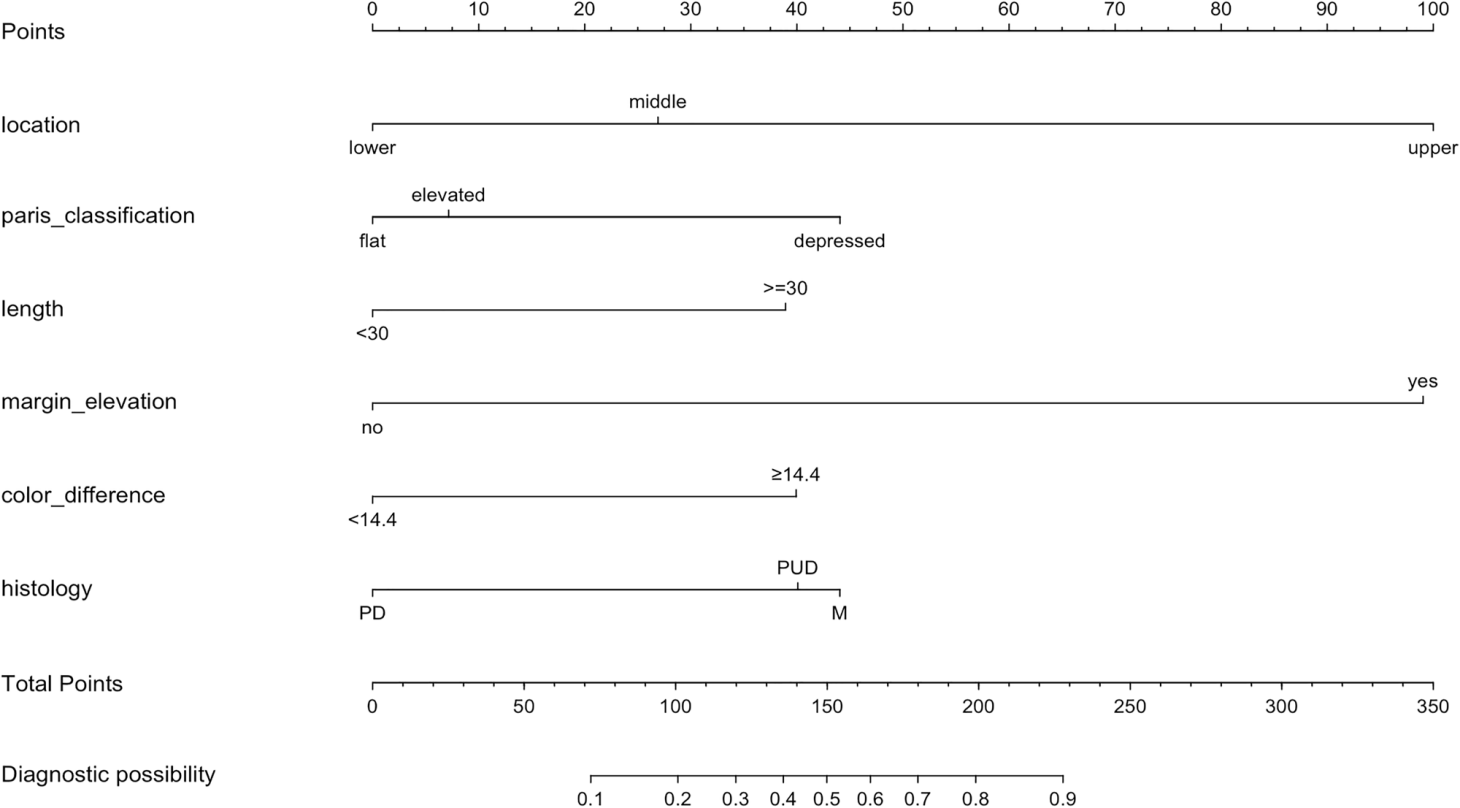
Nomogram for depth invasion prediction in early gastric cancer.

After univariate analysis, 15 factors with a *P* value less than 0.05 were included in stepwise logistic regression in the derivation group, which resulted in 6 variables that were statistically significant predictors of SM invasion (Table 1). These variables include location, macroscopic type, length, marked margin elevation, WLI color difference, histological type. Lesions with the characteristics of depressed macroscopic type, length ≥ 30 mm, marked elevation and larger WLI color difference ≥ 14.4 and mix histological type are more likely to have SM invasion. While lesions located in the middle or lower third of the stomach tend to be intramucosal (mucous layer, M) cancer. Then we developed a nomogram predicting SM invasion of EGC based on logistic regression results (Fig. 4). The AUROC to predict SM invasion in the derivation and validation set was 0.881 and 0.840 respectively.

**Table 1:** Stepwise regression analyses of predictors of SM invasion.

Characteristics		β-coefficient	OR (95%CI)	*P* value
Location	Upper			
	Middle	− 2.05 ± 0.46	0.13 (0.05–0.32)	< .001
	Lower	− 2.78 ± 0.38	0.06 (0.03–0.13)	< .001
Macroscopic type	Elevated			
	Flat			
	Depressed	0.84 ± 0.32	2.32 (1.25–4.32)	.008
Length	< 30			
	≥ 30	1.04 ± 0.34	2.84 (1.46–5.53)	.002
Margin elevation	No			
	Yes	2.72 ± 0.35	15.23 (7.71–30.10)	< .001
WLI color difference ≥ 14.4	No			
	Yes	1.07 ± 0.29	2.91 (1.65–5.16)	< .001
Histology	PD			
	MIX	0.88 ± 0.39	2.41 (1.13–5.13)	.023
	PUD

### Decision tree and random forest models

The decision tree and random forest models were established using a derivation dataset (570 records after the bootstrapping) and evaluated by validation sets (174 records) data. The factors of models were screened based on the feature selection discussed above (*P* < 0.05 in univariate analysis).

**Figure 5:**
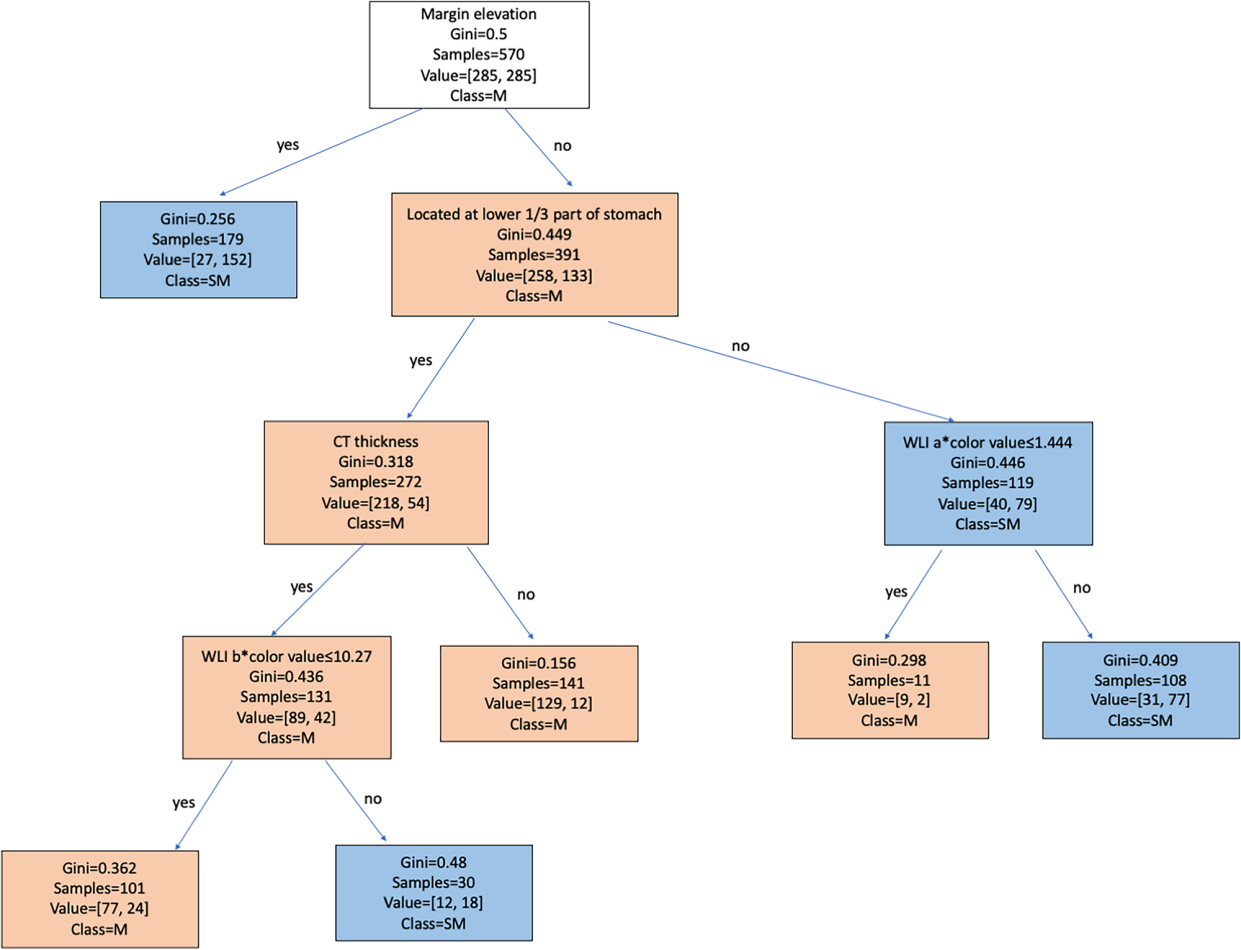
Decision tree model for depth invasion prediction in early gastric cancer. One can start from the root node (margin elevation). If the lesion is categorized as marked margin elevation, then a prediction of SM invasion can be made in the decision tree model. If the lesion is not determined as marked margin elevation, then the next node can be moved to, which is located in the lower 1/3 part of the stomach. Continue comparing the lesion’s characteristics with other internal nodes of the tree until the predicted outcome is reached.

The decision tree model (Fig. 5) has an accuracy of 0.81 in the derivation set and 0.73 in the validation set. The sensitivity and specificity of the derivation set were 0.87 and 0.75 respectively. For the 57 cases with SM invasion in the validation set, the decision tree correctly classified 45 lesions, with a sensitivity of 0.79 and specificity of 0.70 The AUROC of the model for the derivation and validation set were 0.851 and 0.810 respectively.

**Figure 6:**
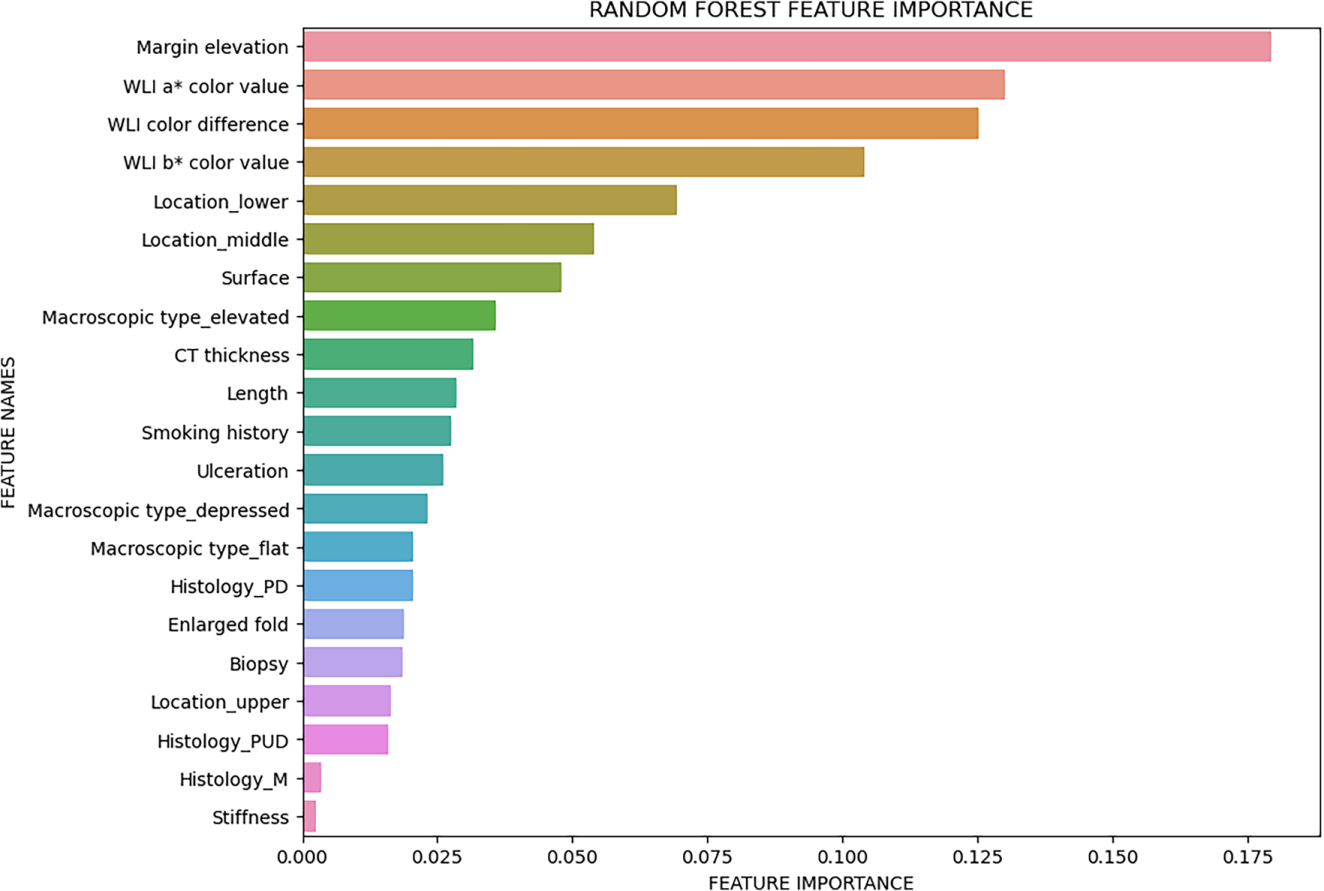
The influence weight of each feature calculated by the random forest model.

**Figure 7:**
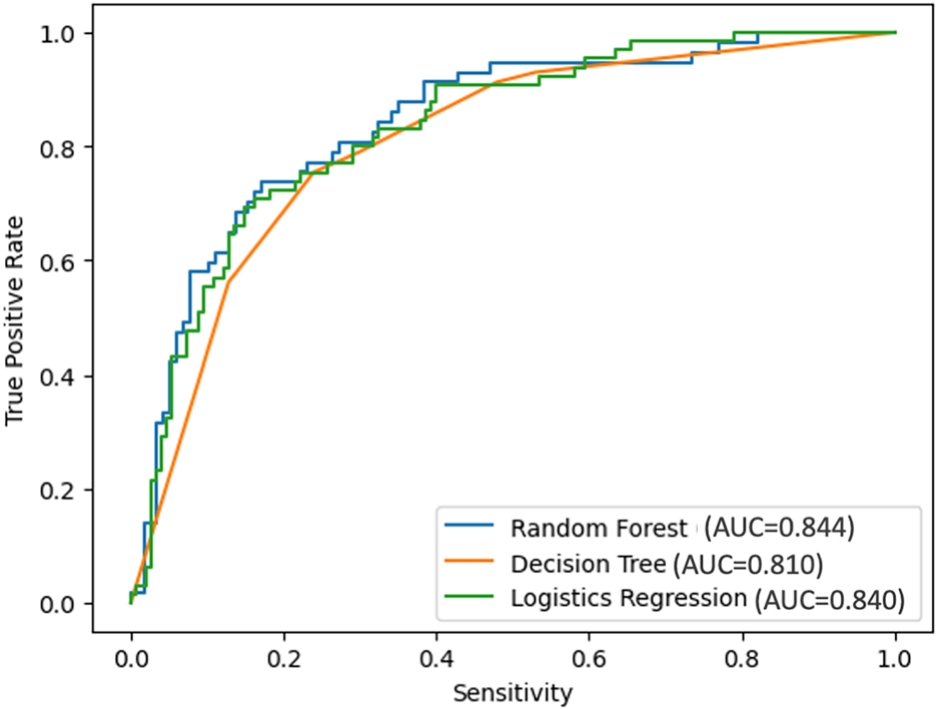
ROC curve of the logistics regression, decision tree model, and random forest model.

For the more sophisticated random forest model (Fig. 6), the derivation dataset and corresponding features are same as decision tree to keep results comparable. The random forest has a sensitivity of 0.75 and a specificity of 0.82 in the validation set, with an AUROC of 0.844. The accuracy of the derivation set and validation set were 0.96 and 0.80 (Fig. 7).

## Discussion

In this study, we retrospectively collected patients’ clinical data and screened for possible influencing independent variables. Among these variables, remarkable redness was one of the features often been subjectively defined as it is likely to bias the observers due to different color and brightness level of surrounding environment under the endoscope. To resolve such challenge, we innovatively used CIE to differentiate chromaticity in this study. To our knowledge, such an approach hasn’t been applied in the depth prediction model of EGC.

Concerning the univariate analysis for all collected independent variables, it demonstrated that the lesion with a larger color difference from the surrounding tissues tends to have a high risk of deeper infiltration. Such finding implies allowing the WLI color metrics to be measured real-time in endoscopic images may improve the diagnostic accuracy of invasion depth of EGC, especially for the less experienced endoscopist. For other predictive features, Abe et al.^2^ and Choi et al.^3^ reported length ≥ 30 as an independent risk factor for a deeper invasion which is in line with our outcomes. Lesions located in the upper 1/3 of the stomach are more inclined to invade into the SM layer^4,17^, considered to be related to the thinner structure of the gastric wall in the upper part of the stomach. In addition, lesions in the upper portion are more difficult to detect in the early stages due to limited viewing angles. Marked margin elevation was considered to be a promising predictor in Abe et al.^2^, Nagahama et al.^13^, and Yao et al.’s research^18^. After the precise definition of the factor, a sensitivity of 92%, a specificity of 97.7%, and an accuracy of 96.9% can be achieved with the single use of the index of marked margin elevation^13^. When the cancer cells infiltrate into the submucosa layer, there is regional stiffness and hypertrophy of the submucosal infiltration site due to the cancer cell mass and fibrosis at the infiltration site, and when the gastric wall is fully extended by air delivery through the endoscope, the submucosal infiltration site does not extend, while the surrounding area extends, showing a margin elevation. Yamada et al.^19^ and Jiang et al.^20^ reported lesions presented as depressed types and mix histologic type were more predisposed to SM invasion and lymph node metastases, which were consistent with our results.

Based on screened variables from univariate analysis, we trained a logistic regression to build a nomogram model as benchmark. The logistic regression model reached an AUROC of 0.840 in the validation set. To improve the prediction accuracy and explore the clinic application, we further studied machine learning algorithms which have better model interpretability. We constructed a decision tree model and a random forest model. Both models prevailed in dealing with non-linear relationships compared with traditional approaches. And to our knowledge, this is the first study focusing on these two types of deep learning models on the depth prediction of EGC. Concerning each model’s clinical implication, the decision tree model’s strong model interpretability allowed designing a straightforward diagnosis procedure; while the random forest model allowed the importance of clinical indicators to be understood. Per our results, the decision tree model built demonstrated margin elevation, lesion located in the lower 1/3 part of the stomach, WLI a*color value, b*color value, and abnormal thickness in enhanced CT were selected. In the random forest model, margin elevation, WLI a* color value, WLI color difference, WLI b* color value, and located in the middle or lower part of the stomach are the six metrics that have the greatest impact on prediction results. The factors screened by all three models developed in this paper are generally in line, only slightly differing in the predictive importance of some variables. Among all three models the random forest prevailed with AUROC equals to 0.844. The machine learning algorithm also suggested that WLI b* color value and enhanced CT could potentially improve prediction accuracy and require further exploration.

Apart from the decision tree model and random forest model mentioned above, scholars have explored the application of CNNs in the depth prediction of EGC as well. Yoon et al.^10^ developed the CNN model with an AUROC of 0.851 using 11,539 endoscopic images. Zhu et al.^12^ and Nagao et al^21^ reported CNN models achieving an AUROC of 0.94 and 0.959, respectively. Goto et al.^9^ have developed a diagnostic method based on endoscopists and an AI classifier, achieving the accuracy of 78.0%, which is higher than through AI classifier or endoscopists alone. Although the AUROC and accuracy of CNN seem to be higher than the decision tree and random forest models studied in this research, the decision-making progress CNN models is more like a black box with poor interpretability. Some scholars have also stated the CNN models show the tendency of over-learning. While the decision tree model provides a clear decision-making process that is easy to follow clinically, and the random forest model visualizes the importance of each feature in the prediction model, both making it easier to understand and apply in different level of medical settings. Current deep learning models of depth prediction of EGC are mainly using static images rather than videos, which differs from clinical setting. Wu et al.^11^ have tried to introduce the real-time videos in the detection of gastric cancer lesions in a deep learning model, reaching a sensitivity of 92.8%. Real-time videos can be further applied to gastric cancer infiltration depth machine learning in future subsequent studies.

The main innovations of this study are as follows: Firstly, we explore multiple variables including clinical characteristics, laboratory tests, CT results, endoscopic characteristics, and pathological results. Among these, we also innovatively introduced CIE in color quantification, which standardized the color metrics and ruled out subjectiveness. The result has significant clinical value, as a plug-in applet can be installed within the endoscopic image system to automatically calculate the color difference between the sites selected by the endoscopist in real-time, therefore indicative to estimating the depth of infiltration while endoscopically observing the patient. Secondly, apart from the logistic regression model, we further introduce machine learning into the study. Systemically screening wide ranges of predictors using decision trees and random forests demonstrated the feature’s importance intuitively. All three models achieved strong prediction results.

The study also has some limitations. Firstly, it is a retrospective single-center study, which resulted in limitations in sample size. We have tried to establish cutoff points for continuous values like WLI a*color, b*color and WLI color difference, but the exact cutoff points of these variables still require future multicenter prospective studies to be further determined. Secondly, historical endoscopic image reading may bring some disparity since some images may have restricted angles. This study retrospectively reads static images, which is still a gap from reading dynamic videos in the actual clinical setting. Due to the limited sample size, some variables have quite data missing, which restricted the introduction of more complex machine learning models. Thirdly, due to the limited number of endoscopically resected submucosal carcinomas, our study included specimens from both surgery and ESD. However, there are some differences in the intervals of resected sections between the processing of these two specimens, which can result in an underestimation of the depth of invasion and affect the efficacy of the prediction model. Future studies with larger samples could try to include only endoscopically or surgically resected specimens for more accurate analysis. In conclusion, the models with color metrics using logistic regression and machine learning algorithms may be useful in making treatment decisions for EGC. Future prospective study and external validation can be performed at multiple centers to further validate the accuracy of the model.

### Supplementary Information

Supplementary Information.

## Data Availability

The datasets generated and/or analyzed in this study are available from the corresponding author upon reasonable request.
