# Supplementary material for: Machine learning models to predict submucosal invasion in early gastric cancer based on endoscopy features and standardized color metrics
Source: Sci Rep. 2024 May 7;14:10445. doi: 10.1038/s41598-024-61258-1 (PMC11076543; doi:10.1038/s41598-024-61258-1)
Supplement: Supplementary file 1 — Supplementary Information. [file 41598_2024_61258_MOESM1_ESM.docx]

**Supplementary Table S1** Characteristics of the lesions in the derivation and validation sets

| Characteristics | Derivation set(n=504) | validation set(n=214) | *P* value |
| --- | --- | --- | --- |
| Age |  |  | .113 |
| <60 | 176 (34.9%) | 61 (28.5%) |  |
| >=60 | 328 (65.1%) | 153 (71.5%) |  |
| Gender |  |  | .893 |
| female | 153 (30.4%) | 63 (29.6%) |  |
| male | 350 (69.6%) | 150 (70.4%) |  |
| Alcohol consumption |  |  | 1.000 |
| no | 351 (69.8%) | 149 (69.6%) |  |
| yes | 152 (30.2%) | 65 (30.4%) |  |
| Smoking history |  |  | .128 |
| no | 337 (67.0%) | 130 (60.7%) |  |
| yes | 166 (33.0%) | 84 (39.3%) |  |
| Gastric cancer history |  |  | .405 |
| no | 453 (90.2%) | 198 (92.5%) |  |
| yes | 49 (9.76%) | 16 (7.48%) |  |
| Neutrophils | 3.81 (4.28) | 3.70 (1.72) | .638 |
| Lymphocytes | 1.81 (3.57) | 1.62 (0.56) | .234 |
| Monocyte | 0.37 (0.14) | 0.39 (0.15) | .092 |
| Platelet | 205 (55.3) | 199 (54.6) | .253 |
| Hemoglobin | 137 (19.1) | 141 (31.2) | .094 |
| Albumin | 42.5 (3.87) | 42.0 (4.16) | .161 |
| Location |  |  | .782 |
| upper 1/3 | 116 (23.0%) | 52 (24.3%) |  |
| middle 1/3 | 88 (17.5%) | 33 (15.4%) |  |
| lower 1/3 | 300 (59.5%) | 129 (60.3%) |  |
| Enlarged fold |  |  | .345 |
| no | 471 (93.5%) | 195 (91.1%) |  |
| yes | 33 (6.55%) | 19 (8.88%) |  |
| Ulceration |  |  | .058 |
| no | 338 (67.1%) | 127 (59.3%) |  |
| yes | 166 (32.9%) | 87 (40.7%) |  |
| Macroscopic type |  |  | .032 |
| elevated | 243(48.4%) | 94(43.9%) |  |
| flat | 77(15.3%) | 22(10.3%) |  |
| depressed | 182(36.3%) | 98(45.8%) |  |
| Length |  |  | .611 |
| <30mm | 392(77.8%) | 162(75.7%) |  |
| ≥30mm | 112(22.2%) | 52(24.3%) |  |
| Spontaneous bleeding |  |  | .138 |
| no | 472(93.7%) | 207(96.7%) |  |
| yes | 32(6.35%) | 7(3.27%) |  |
| Central depression |  |  | .276 |
| no | 415(82.3%) | 184(86.0%) |  |
| yes | 89(17.7%) | 30(14.0%) |  |
| Stiffness |  |  | .069 |
| no | 486(82.3%) | 199(93.0%) |  |
| yes | 18(3.57%) | 15(7.01%) |  |
| Margin elevation |  |  | .419 |
| no | 397(78.8%) | 162(75.7%) |  |
| yes | 107(21.2%) | 52(24.3%) |  |
| Demarcation |  |  | .656 |
| unclear | 81(16.1%) | 38(17.8%) |  |
| clear | 423(83.9%) | 176(86.2%) |  |
| surface |  |  | .678 |
| smooth | 263(52.2%) | 116(54.2%) |  |
| nodular | 241(47.8%) | 98(45.8%) |  |
| Depth |  |  | .998 |
| M | 354(70.24%) | 149(69.6%) |  |
| SM | 150(29.8%) | 65(30.4%) |  |
| Histology |  |  | .211 |
| PD | 391 (77.7%) | 168 (78.5%) |  |
| PUD | 86 (17.1%) | 29 (13.6%) |  |
| M | 26 (5.17%) | 17 (7.94%) |  |
| Biopsy |  |  | .811 |
| Ca | 410 (82.5%) | 178 (83.6%) |  |
| ATP | 87 (17.5%) | 35 (16.4%) |  |
| Atrophy |  |  | 0.956 |
| no | 26(5.2%) | 11(5.1%) |  |
| mild | 285(56.8%) | 118(55.1%) |  |
| medium | 52(10.4%) | 25(11.7%) |  |
| severe | 139(27.7%) | 60(28.0%) |  |
| CT thickness |  |  | .900 |
| no | 213 (50.1%) | 88 (49.2%) |  |
| yes | 212 (49.9%) | 91 (50.8%) |  |
| CT enlarged lymph nodes |  |  | .836 |
| no | 363 (85.4%) | 151 (84.4%) |  |
| yes | 62 (14.6%) | 28 (15.6%) |  |
| WLI color difference | 13.5 (10.9) | 12.2 (7.16) | .061 |
| WLI a* color value | 9.06 (11.4) | 8.87 (6.92) | .787 |
| WLI b* color value | 6.90 (6.52) | 6.21 (5.99) | .176 |

**Supplementary Table S2.** Univariate analyses of predictors of SM invasion

| Characteristics | |  | M (N=402) | SM (N=181) | OR (univariable) | *P* value | |
| --- | --- | --- | --- | --- | --- | --- | --- |
| Age | <60 | | 137 (34.1%) | 59 (32.6%) |  |  |  |
|  | >=60 | | 265 (65.9%) | 122 (67.4%) | 1.07 (0.74-1.55) | .726 |  |
| Gender | female | | 128 (31.8%) | 46 (25.4%) |  |  |  |
|  | male | | 274 (68.2%) | 135 (74.6%) | 1.37 (0.92-2.04) | .117 |  |
| Alcohol consumption | no | | 285 (70.9%) | 121 (66.9%) |  |  |  |
|  | yes | | 117 (29.1%) | 60 (33.1%) | 1.21 (0.83-1.76) | .326 |  |
| Smoking history | | no | 276 (68.7%) | 104 (57.5%) |  |  |  |
|  | | yes | 126 (31.3%) | 77 (42.5%) | 1.62 (1.13-2.33) | .009 |  |
| Gastric cancer history | | no | 364 (90.5%) | 166 (91.7%) |  |  |  |
|  | | yes | 38 (9.5%) | 15 (8.3%) | 0.87 (0.46-1.62) | .651 |  |
| Neutrophils | | Mean ± SD | 3.6 ± 1.4 | 3.7 ± 2.0 | 1.04 (0.94-1.16) | .432 |  |
| Lymphocytes | | Mean ± SD | 1.6 ± 0.6 | 2.1 ± 5.9 | 1.13 (0.84-1.51) | .427 |  |
| Monocytes | | Mean ± SD | 0.4 ± 0.1 | 0.4 ± 0.1 | 2.67 (0.77-9.26) | .121 |  |
| Platelets | | Mean ± SD | 200.8 ± 53.9 | 206.9 ± 54.2 | 1.00 (1.00-1.01) | .210 |  |
| Hemoglobin | | Mean ± SD | 138.7 ± 21.4 | 138.1 ± 21.4 | 1.00 (0.99-1.01) | .751 |  |
| Albumin | | Mean ± SD | 42.4 ± 3.9 | 42.5 ± 3.8 | 1.01 (0.96-1.05) | .782 |  |
| Location | | Upper 1/3 | 63 (15.7%) | 65 (35.9%) |  |  |  |
|  | | Middle 1/3 | 63 (15.7%) | 35 (19.3%) | 0.54 (0.31-0.92) | .025 |  |
|  | | Lower 1/3 | 276 (68.7%) | 81 (44.8%) | 0.28 (0.19-0.44) | <.001 |  |
| Macroscopic type | | elevated | 261 (51.9%) | 76 (35.5%) |  |  |  |
|  | | flat | 84 (16.7%) | 16 (7.5%) | 0.65 (0.36-1.18) | .160 |  |
|  | | depressed | 158 (31.4%) | 122 (57%) | 2.64 (1.87-3.76) | <.001 |  |
| Length | | <30mm | 328 (81.6%) | 125 (69.1%) |  |  |  |
|  | | ≥30mm | 74 (18.4%) | 56 (30.9%) | 1.99 (1.33-2.97) | <.001 |  |
| Spontaneous bleeding | | no | 378 (94%) | 176 (97.2%) |  |  |  |
|  | | yes | 24 (6%) | 5 (2.8%) | 0.45 (0.17-1.19) | .108 |  |
| Central depression | | no | 335 (83.3%) | 156 (86.2%) |  |  |  |
|  | | yes | 67 (16.7%) | 25 (13.8%) | 0.80 (0.49-1.32) | .382 |  |
| Stiffness | | no | 388 (96.5%) | 164 (90.6%) |  |  |  |
|  | | yes | 14 (3.5%) | 17 (9.4%) | 2.87 (1.38-5.96) | .005 |  |
| Margin elevation | | no | 360 (89.6%) | 84 (46.4%) |  |  |  |
|  | | yes | 42 (10.4%) | 97 (53.6%) | 9.90 (6.42-15.26) | <.001 |  |
| Demarcation | | unclear | 71 (17.7%) | 22 (12.2%) |  |  |  |
|  | | clear | 331 (82.3%) | 159 (87.8%) | 1.55 (0.93-2.59) | .095 |  |
| surface | | smooth | 227 (56.5%) | 70 (38.7%) |  |  |  |
|  | | nodular | 175 (43.5%) | 111 (61.3%) | 2.06 (1.44-2.94) | <.001 |  |
| Enlarged fold | | no | 382 (95%) | 152 (84%) |  |  |  |
|  | | yes | 20 (5%) | 29 (16%) | 3.64 (2.00-6.64) | <.001 |  |
| Ulceration | | no | 281 (69.9%) | 81 (44.8%) |  |  |  |
|  | | yes | 121 (30.1%) | 100 (55.2%) | 2.87 (2.00-4.12) | <.001 |  |
| Histology | | PD | 323 (80.3%) | 119 (65.7%) |  |  |  |
|  | | PUD | 65 (16.2%) | 38 (21%) | 1.59 (1.01-2.49) | .045 |  |
|  | | MIX | 14 (3.5%) | 24 (13.3%) | 4.65 (2.33-9.29) | <.001 |  |
| Biopsy | | Ca | 324 (80.6%) | 169 (93.4%) |  |  |  |
|  | | ATP | 78 (19.4%) | 12 (6.6%) | 0.29 (0.16-0.56) | <.001 |  |
| Atrophy | | no | 21(4.2%) | 16(7.4%) |  |  |  |
|  | | mild | 292(58.2%) | 113(52.6%) | 0.51(0.26-1.01) | .053 |  |
|  | | medium | 46(9.2%) | 31(14.4%) | 0.88(0.40-1.96) | .762 |  |
|  | | severe | 143(28.5%) | 55(25.6%) | 0.50(0.25-1.04) | .063 |  |
| CT thickness^*^ | | no | 232 (57.7%) | 64 (35.4%) |  |  |  |
|  | | yes | 170 (42.3%) | 117 (64.6%) | 2.49 (1.73-3.59) | <.001 |  |
| CT enlarged lymph nodes^*^ | | no | 348 (86.6%) | 146 (80.7%) |  |  |  |
|  | | yes | 54 (13.4%) | 35 (19.3%) | 1.54 (0.97-2.46) | .068 |  |
| WLI color difference | | Mean ± SD | 12.6 ± 10.9 | 15.5 ± 8.6 | 1.03 (1.01-1.06) | .003 |  |
| WLI a*color value | | Mean ± SD | 8.1 ± 11.6 | 11.8 ± 7.9 | 1.06 (1.04-1.09) | <.001 |  |
| WLI b*color value | | Mean ± SD | 6.5 ± 6.0 | 8.1 ± 6.9 | 1.04 (1.01-1.07) | .005 |  |


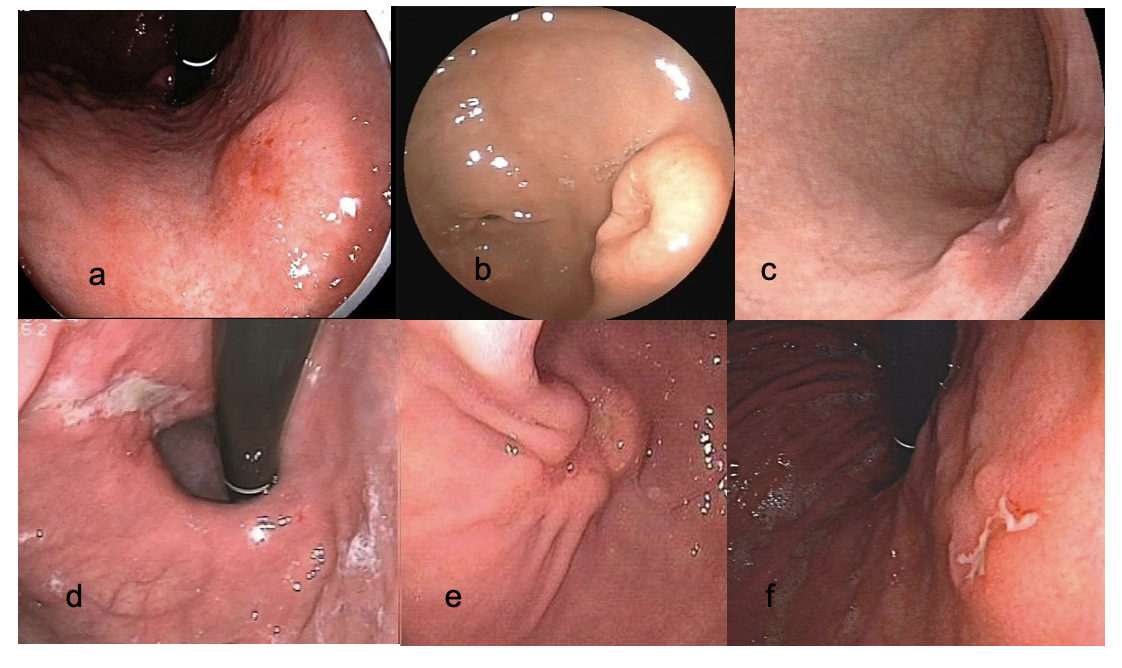


Supplementary Figure 1 Marked margin elevation (1a-1c): (1) a manifestation of elevation of the lesion itself as a trapezoid elevation or (2) mucosal folds converged and were elevated at the lesion site when viewed from a distance of 15 to 45 degrees under conditions of full extension of the gastric wall. Ulceration(1d): endoscopic picture of ulceration. Enlarged folds (1e): thickened or merged folds toward inside of the lesion. Nodular surface (1f): uneven surface
